# Engineered nanomaterials: exposures, hazards, and risk prevention

**DOI:** 10.1186/1745-6673-6-7

**Published:** 2011-03-21

**Authors:** Robert A Yokel, Robert C MacPhail

**Affiliations:** 1 Department of Pharmaceutical Sciences, College of Pharmacy and Graduate Center for Toxicology, University of Kentucky, Lexington, KY, 40536-0082, USA; 2 Toxicity Assessment Division, National Health and Environmental Effects Research Laboratory, U.S. Environmental Protection Agency, Research Triangle Park NC, 27711, USA

## Abstract

Nanotechnology presents the possibility of revolutionizing many aspects of our lives. People in many settings (academic, small and large industrial, and the general public in industrialized nations) are either developing or using engineered nanomaterials (ENMs) or ENM-containing products. However, our understanding of the occupational, health and safety aspects of ENMs is still in its formative stage. A survey of the literature indicates the available information is incomplete, many of the early findings have not been independently verified, and some may have been over-interpreted. This review describes ENMs briefly, their application, the ENM workforce, the major routes of human exposure, some examples of uptake and adverse effects, what little has been reported on occupational exposure assessment, and approaches to minimize exposure and health hazards. These latter approaches include engineering controls such as fume hoods and personal protective equipment. Results showing the effectiveness - or lack thereof - of some of these controls are also included. This review is presented in the context of the Risk Assessment/Risk Management framework, as a paradigm to systematically work through issues regarding human health hazards of ENMs. Examples are discussed of current knowledge of nanoscale materials for each component of the Risk Assessment/Risk Management framework. Given the notable lack of information, current recommendations to minimize exposure and hazards are largely based on common sense, knowledge by analogy to ultrafine material toxicity, and general health and safety recommendations. This review may serve as an overview for health and safety personnel, management, and ENM workers to establish and maintain a safe work environment. Small start-up companies and research institutions with limited personnel or expertise in nanotechnology health and safety issues may find this review particularly useful.

## 1. Introduction

### A. The objectives of this review

Although there has been considerable work to advance nanotechnology and its applications, understanding the occupational, health and safety aspects of engineered nanomaterials (ENMs) is still in its formative stage. The goals of this review are to describe some general features of ENMs, how a worker might be exposed to ENMs, some potential health effects, and approaches to minimize exposure and toxicity. The target audience includes industrial hygienists, investigators working with these materials, institutes and universities conducting research, and start-up companies that may not have the necessary occupational health and safety expertise, knowledge, and/or staff.

A comprehensive review described the field of nanotoxicology six years ago, including some mechanisms of toxicity, portals of ENM entry, their translocation, and the state of their risk assessment at the time [1]. More recent reviews have focused on the major challenges, key questions, and research needs to assess ENM toxicity and risk [2-7]. This review addresses issues not extensively covered in prior reviews, including recent exposure-assessment studies, and engineering and personal protective equipment (PPE) options and their efficacy to minimize ENM exposure. This review also includes accepted but not yet published reports, recently completed studies not yet published, and ongoing work. Our goal was to provide up-to-date information on ENM exposures, their health hazards, and ways to minimize risk.

### B. Engineered nanomaterials

Nano is a prefix derived from the Greek word for dwarf. The parts of the U. S. National Nanotechnology Initiative (NNI) definition that are relevant for this review define nanoscale materials as having at least one dimension in the range of 1 to 100 nanometers (nm), with properties that are often unique due to their dimensions, and that are intentionally manufactured [8]. There are many definitions of nanoscale materials, which generally encompass the same bounds on ENM size [9,10]. This is in contrast to naturally occurring and unintentionally-produced materials on the same scale, which are referred to as ultrafine particles. The term ultrafine has been used by the aerosol research and occupational and environmental health communities to describe airborne particles smaller than 100 nm in diameter [11]. Ultrafine particles are not intentionally produced. They are the products of combustion and vaporization processes such as welding, smelting, fuel combustion, fires, and volcanoes [1,12,13]. In this review, intentionally-manufactured nanoscale materials will be referred to as ENMs. They are usually produced by bottom-up processes, such as physical and chemical vapor deposition, liquid phase synthesis, and self-assembly [5,14].

The health and environmental effects of ENMs are not well understood, leading some to caution development of this technology [15-19]. Some understanding of ENM effects can be derived, however, by analogy from ultrafine particles, which have been shown to produce inflammation, exacerbation of asthma, genotoxicity, and carcinogenesis following inhalation. The following sections describe ENMs, and some of their uses and uncertainties, providing the context of this review.

### C. Common ENM size, composition, and quality

**Figure 1:**
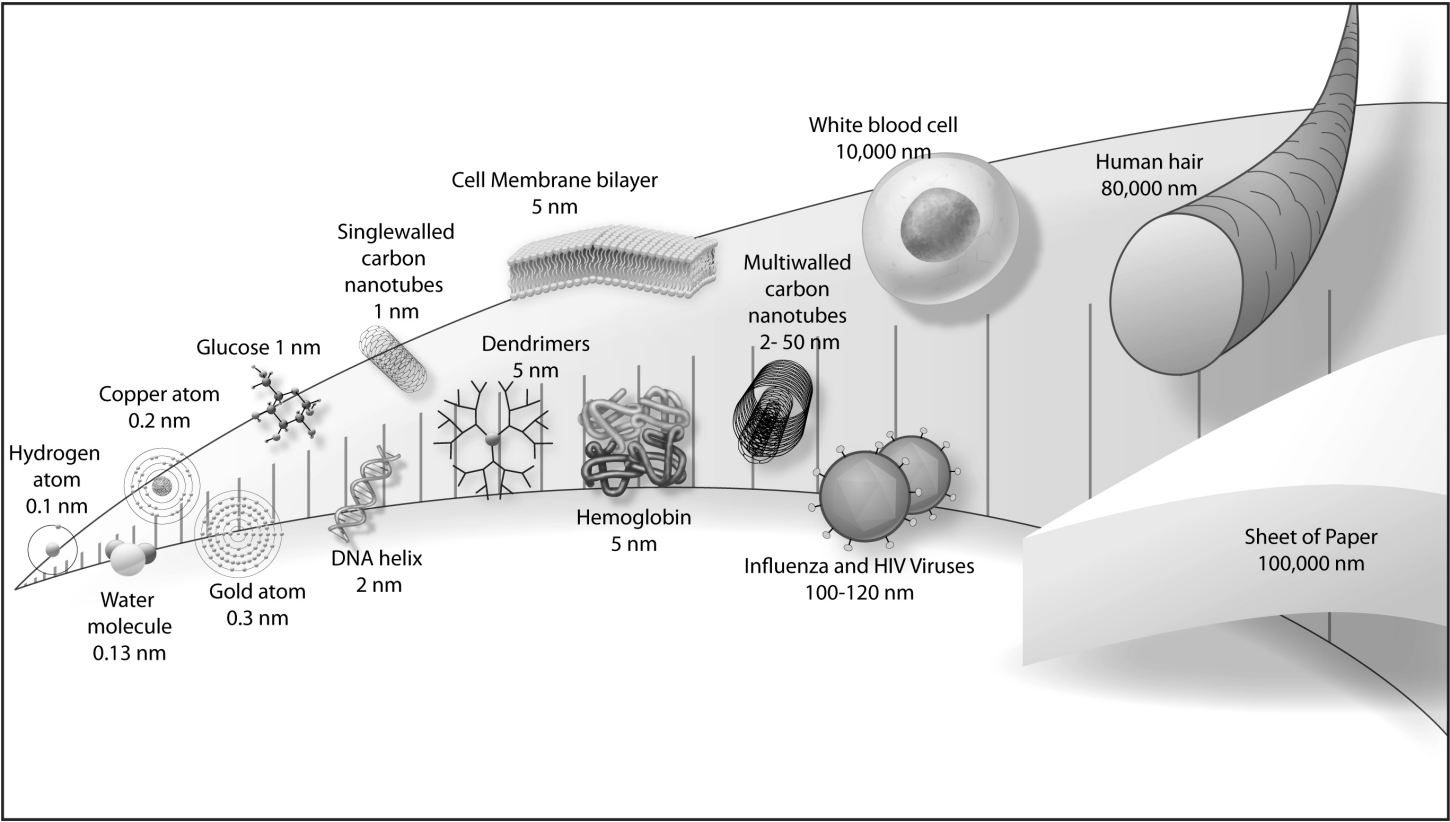
**The sizes and shapes of some ENMs compared to more familiar materials**. Shown for comparison are materials that are below, within, and above the nanoscale range, to put ENM size in perspective.

Figure 1 relates ENM size to other chemical and biological materials. There are a staggering number of ENM compositions and shapes. Over 5000 patents have been issued for carbon nanotubes (CNTs) and > 50,000 varieties of CNTs have been produced [20]. The sheer number of ENMs contributes to the lack of our adequate understanding of ENM health and safety. They are primarily composed of carbon or metal/metal oxide, as illustrated by the representative manufactured nanomaterials selected for testing by the Organisation for Economic Co-operation and Development (OECD) [21]. Carbon-based ENMs include single-walled and multi-walled carbon nanotubes (SWCNTs and MWCNTs), graphene (a single sheet of carbon atoms in a hexagonal structure), spherical fullerenes (closed cage structures composed of 20 to 80 carbon atoms consisting entirely of three-coordinate carbon atoms, e.g., C_60 _[Buckyballs, buckminsterfullerene]), and dendrimers, which are symmetrical and branched. SWCNTs and MWCNTs are ~1 to 2 and 2 to 50 nm wide, respectively, and can be > 1 μm long. The C_60 _diameter is ~1 nm. Metal and metal oxide ENMs most commonly studied are cadmium in various complexes, gallium arsenide, gold, nickel, platinum, silver, aluminum oxide (alumina), cerium dioxide (ceria), silicon dioxide (silica), titanium dioxide (TiO_2_, titania), and zinc oxide. The size of ENMs is in the same range as major cellular machines and their components, such as enzymes, making it likely that they will easily interact with biochemical functions [22].

Some ENMs contain contaminants, such as residual metal catalysts used in the synthesis of CNTs. ENM toxicity has been attributed to these residual metals, as discussed in **II, B, 1**. **ENM exposure effects in the lung**. The physico-chemical properties of ENMs, when tested prior to their use, are often different from those stated by the supplier [23,24]. A major cause of changes in the physico-chemical properties of ENMs over time and in various media is agglomeration, discussed in **II, A, 2. The physico-chemical properties of ENMs that impact their uptake**. When ENMs are not sufficiently characterized to identify their composition or properties it makes the prediction of toxicity, when added to the insufficient understanding of their biological effects, even more difficult [25].

### D. Some uses of ENMs and the projected market and workforce

There is considerable interest in developing ENMs because their properties differ in fundamental and valuable ways from those of individual atoms, molecules, and bulk matter. Nanoscale products and materials are increasingly being used in optoelectronic, electronic (e.g., computer hard drives), magnetic, medical imaging, drug delivery, cosmetic and sunscreen, catalytic, stain resistant fabric, dental bonding, corrosion-resistance, and coating applications [26]. Major future applications are expected to be in motor vehicles, electronics, personal care products and cosmetics, and household and home improvement. These applications capitalize on their electromagnetic, catalytic, pharmacokinetic, and physico-chemical properties, including strength, stiffness, weight reduction, stability, anti-fogging, and scratch resistance. Current products contain various ENMs including nanotubes, metal oxides, and quantum dots (semiconductors developed as bright, photostable fluorescent dyes and imaging agents). Nanowerk identified ~2500 commercial nanomaterials, including ~27% metal oxides, 24% CNTs, 18% elements, 7% quantum dots, and 5% fullerenes [http://www.nanowerk.com/phpscripts/n_dbsearch.php]. There are > 1000 consumer products available that contain ENMs. They are primarily composed of silver, carbon, zinc, silica, titania and gold. The main application is in health and fitness products [27,28]. Three to four new nanotechnology-containing consumer products are introduced weekly into the market, according to The Project on Emerging Nanotechnologies [http://www.nanotechproject.org/inventories/consumer/].

The anticipated benefits of ENM applications resulted in expenditure of $18 billion worldwide on nanotechnology research and development in 2008. In 2004 Lux Research predicted that nanotechnology applications will become commonplace in manufactured goods starting in 2010 and become incorporated into 15% of global manufacturing output in 2014 [https://portal.luxresearchinc.com/research/document_excerpt/2650]. The ENM workforce is estimated to grow ~15% annually [29]. An epidemiological feasibility study of CNT workers initiated in 2008 revealed most manufacturers were small companies that had no environmental/occupational health and safety person and little knowledge about this topic [30]. By 2015, the global market for nanotechnology-related products is predicted to employ 2 million workers (at least 800,000 in the U.S.) to support nanotechnology manufacturing, and $1 trillion in sales of nanotechnology-related products [31].

### E. Uncertainties regarding the adverse effects of ENMs

There have been concerns about the safety and public acceptance of this burgeoning technology, particularly in the past 5 years, due to the lack of much information about potential adverse effects [32]. This resulted in an increase from 2.9 to 6.6% of the NNI budget for environmental health and safety from 2005 to 2011. Prior to 2005 it does not seem funds were specifically allocated for this purpose nor was the U.S. National Institute for Occupational Safety and Health (NIOSH) a contributor to NNI funding [33,34]. The United Nations Educational, Scientific and Cultural Organization (UNESCO) compared the concerns of the public over new products with their perception of genetically modified foods/organisms to nanotechnology. They noted that the lack of knowledge can result in restrictions, outright bans, and international conflicts over production, sale, and transport of such materials [35]. Public acceptance can influence the success of an emergent technology, as public opinion is considerably influenced by information prior to the adoption of the technology. However, individuals form opinions often when they do not possess much information, based on factors other than factual information, including values, trust in science, and arguments that typically lack factual content [36]. This creates a challenge to earn public acceptance of nanotechnology.

There is a notable lack of documented cases and research of human toxicity from ENM exposure. It is widely recognized that little is known about ENM safety. An uncertainty analysis revealed knowledge gaps pervade nearly all aspects of ENM environmental health and safety [4]. Owing to their small size and large surface area, ENMs may have chemical, physical, and biological properties distinctly different from, and produce effects distinct from or of a different magnitude than, fine particles of similar chemical composition. This is discussed in **II, A, 2. The physico-chemical properties of ENMs that impact their uptake**. ENM properties often differ from individual atoms, molecules, and from bulk matter. These differences include a high rate of pulmonary deposition, the ability to travel from the lung to systemic sites, and a high inflammatory potential [1]. Further contributing to our lack of understanding of the potential health effects of ENMs is that most production is still small scale. As such, potential adverse effects from the anticipated increase in large scale production and marketing of ENM-containing products and use are generally unknown. Furthermore, the number of novel ENMs being created continues to grow at a high rate, illustrated by the accelerating rate of nanotechnology-related patent applications [37,38].

## II. A Framework for Evaluating the Risk of ENMs

**Figure 2:**
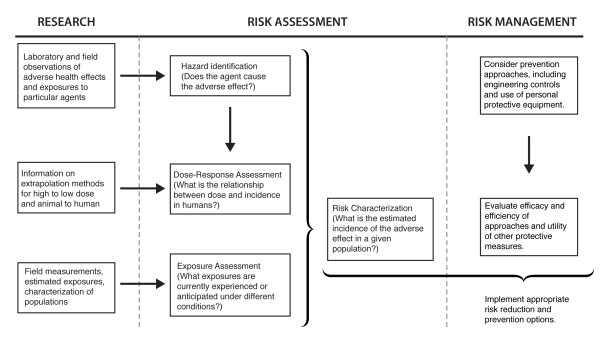
**The Risk Assessment/Risk Management framework**. Modified from [39].

We elected to review the existing literature on ENM effects in the context of the Risk Assessment/Risk Management framework as originally described in the U.S. National Research Council report "Risk Assessment in the Federal Government: Managing the Process", often called the Red Book, that mainly dealt with chemical threats to health [39]. The framework is depicted in Figure 2. A similar approach was advanced by the European Chemicals Bureau for biocidal products (http://eur-lex.europa.eu/pri/en/oj/dat/2003/l_307/l_30720031124en00010096.pdf). Although the NRC framework is portrayed as a sequential approach, in practice it is dynamic with considerable interaction between risk assessors, scientists, and often times the affected parties. This general approach has been proposed for evaluating the risks of ENMs [5-7]. A notable alternative is the Nano Risk framework, a joint venture of the Environmental Defense Fund and DuPont [40]. In addition, due to the many different ENMs, and the time and cost to thoroughly assess their potential risks [41], there is currently much interest in developing *in vitro *models that are predictive of *in vivo *effects [42], although these are not always successful [42-44], and in developing tiered testing systems [45,46]. Additional efforts are underway to group (band) similar ENMs in order to promote safe handling and use of ENMs, and restrict worker exposure, in the absence of definitive health and safety information [47,48]. Still others are applying computational approaches to predict ENM effects, including toxicity [49,50].

In this review the Risk Assessment/Risk Management framework will be used as a template because it succinctly codifies the diverse practices of risk assessment into a logical framework that collects data to determine (1) whether an agent causes an adverse effect, (2) how the effect is related to dose, (3) whether exposure is likely, and (4) the probability of adverse effects in the population at current exposure levels. The framework also embraces research that feeds each of the elements of the risk assessment with the necessary information. For the current review, this framework provides a systematic method to work through the many issues surrounding the potential health effects of ENMs.

The first element, hazard identification, addresses whether there is any evidence that an agent causes an adverse effect. Hazard identification represents the lowest hurdle in the process, since the evidence could come from any number of sources, including laboratory or field observations, and might only be suggestive. The next element, dose-response assessment, is more rigorous and asks whether there is a relationship between the dose of the agent and the incidence or magnitude of adverse effect. This element is based on the fundamental tenet in toxicology and pharmacology of dose response; that is, as the dose increases so does the effect. This information is often not directly available for humans, so laboratory animal studies are typically used. Exposure assessment is the next element. If evidence indicates an agent poses a hazard, and the hazard is dose-related, the next step is to determine the extent of occupational or daily life exposure. Information from all elements is then combined into a risk characterization, which estimates the likelihood of an adverse effect occurring in the exposed population or a segment of the population.

The Risk Assessment/Risk Management framework is comprised of 3 essential components; research, risk assessment, and risk management. Risk assessment is regarded as a scientific undertaking whereas risk management uses the science to regulate exposure to the agent in ways that take into account social benefits, economic costs, and legal precedents for action.

The following sections are arranged to follow the NRC paradigm. Examples are given of adverse effects of ENMs to show why there may be reason for concern. Reports on exposure levels, the likelihood of adverse effects resulting from exposure, and options for minimizing risk are also summarized. This is not, however, an all-inclusive review of the literature; interested readers are referred to the reference section for a number of comprehensive reviews of many of the topics pertaining to ENMs and their effects.

### A. Hazard identification

In the occupational context, hazard identification can be re-stated as "What effects do ENMs have on workers' health?" to which NIOSH has stated: "No conclusive data on engineered nanoparticles exist for answering that question, yet. Workers within nanotechnology-related industries have the potential to be exposed to uniquely engineered materials with novel sizes, shapes, and chemical properties, at levels far exceeding ambient concentrations...much research is still needed." [http://www.cdc.gov/niosh/topics/nanotech/about.html].

Information about ENMs might be obtained from well-documented retrospective analyses of unintended exposures. The most extensive exposures to ENMs likely occur in the workplace, particularly research laboratories; start-up companies; pilot production facilities; and operations where ENMs are processed, used, disposed, or recycled [51]. Occupational hygienists can contribute to the knowledge and understanding of ENM safety and health effects by thorough documentation of exposures and effects. In the U.S., NIOSH is responsible for conducting research and making recommendations for the prevention of work-related illnesses and injuries, including ENMs. The U.S. Occupational Safety and Health Administration (OSHA) is responsible for making and enforcing the regulations.

#### 1. The key routes of ENM exposure

**Figure 3:**
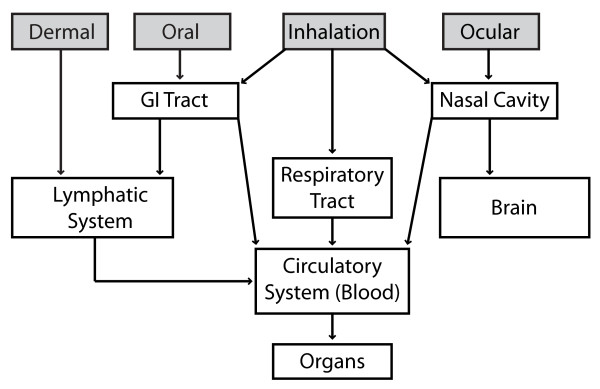
**The predominant routes of ENM exposure and uptake, and potential routes of ENM translocation**. The four gray shaded boxes indicate the primary routes of ENM exposure. The arrows down from these uptake sites show potential translocation pathways. The translocation pathways are described in more detail in **Section II, D. Clearance of ENMs, their translocation to distal sites, and persistence.** For example, the lung might be the primary route of exposure or might be a distal site after uptake from another route and translocation to the lung. ENMs might enter the brain from the nasal cavity or from blood, across the blood-brain barrier.

Figure 3 illustrates the four routes that are most likely to result in ENM exposure of the five organ systems which are the major portals of ENM entry: skin, gastrointestinal tract, lung, nasal cavity, and eyes [22]. It also illustrates the most likely paths of translocation (re-distribution or migration), enabling ENMs to reach organs distal to the site of uptake.

The inhalation route has been of greatest concern and the most studied, because it is the most common route of exposure to airborne particles in the workplace. The skin has also been investigated. Most studies have shown little to no transdermal ENM absorption. Oral (gastrointestinal) exposure can occur from intentional ingestion, unintentional hand-to-mouth transfer, from inhaled particles > 5 μm that are cleared via the mucociliary escalator, and of drainage from the eye socket via the nasal cavity following ocular exposure. Direct uptake of nanoscale materials from the nasal cavity into the brain via the olfactory and trigeminal nerves has been shown. Each of these routes is discussed in more detail below.

Routes that avoid first-pass clearance and metabolism in the gastrointestinal tract and liver include uptake (absorption) from the nasal cavity (either into systemic circulation or directly into the brain), orotransmucosal (e.g., buccal [from the cheek] and sub-lingual), and transdermal. These routes may present a greater risk of ENM-induced adverse effects because more ENM is likely to reach the target organ(s) of toxicity.

#### 2. The physico-chemical properties of ENMs that impact their uptake

Hazard identification has revealed that the physico-chemical properties of ENMs can greatly influence their uptake. ENMs show greater uptake and are more biologically active than larger-sized particles of the same chemistry, due to their greater surface area per mass [52,53]. Additional ENM characteristics that may influence their toxicity include size, shape, surface functionalization or coating, solubility, surface reactivity (ability to generate reactive oxidant species), association with biological proteins (opsonization), binding to receptors, and, importantly, their strong tendency to agglomerate. An agglomeration is a collection of particles that are loosely bound together by relatively weak forces, including van der Waals forces, electrostatic forces, simple physical entanglement, and surface tension, with a resulting external surface area similar to the sum of the surface area of the individual components [9,54]. Agglomeration is different from aggregation. Aggregated particles are a cohesive mass consisting of particulate subunits tightly bound by covalent or metallic bonds due to a surface reconstruction, often through melting or annealing on surface impact, and often having an external surface area significantly smaller than the sum of calculated surface areas of the individual components [9,54]. Agglomerates may be reversible under certain chemical/biological conditions whereas an aggregate will not release primary particles under normal circumstances of use or handling. Airborne ENMs behave very much like gas particles. They agglomerate in air due to self-association (in one study increasing from 8 to 15 nm in 16 min and to ~100 nm in 192 min) and interaction with background aerosols (to ~500 nm agglomerates within min) [55]. Studies of ENMs in occupational settings showed airborne particulates were most commonly 200 to 400 and 2000 to 3000 nm [51,56]. ENMs also agglomerate in liquids, resulting in micrometer sized particles [57]. One study showed that concentration and smaller ENM size positively correlated with speed of agglomeration [58]. Changes in ENM surface area can profoundly uptake and effects.

The aspect ratio (length:diameter) of ENMs also plays a major role in their toxic potential. Particles with a length > 5 μm and aspect ratio ≥ 3:1 are conventionally defined as fibers [59]. Inhaled asbestos containing high aspect-ratio fibers is more toxic than lower aspect-ratio fibers. Foreign materials are often cleared by macrophage phagocytosis, but when too large to be phagocytosed they are not effectively cleared from the lung. This results in release of inflammatory mediators, discussed below.

It appears that ~15 to 30 nm is a critical width or diameter for ENMs to have properties different from the solution and bulk chemistry of their components. Reactive oxygen species generation in an acellular system to which 4 to 195 nm titania ENMs were added was negligible up to 10 nm, then increased up to ~30 nm, when it reached a plateau [53]. A review concluded there is a critical size for ENMs at which new properties typically appear. These new properties are strongly related to the exponential increase in the number of atoms localized at the surface, making metal and metal oxide ENMs with diameters < 20 to 30 nm most different from bulk material [60]. For example, 1 and 3 nm gold ENMs, which contain ~30 and 850 atoms, have nearly all and ~50% of their atoms surface exposed, respectively. Additionally, the optimal particle radius to accelerate adhesion to a cell-surface lipid bilayer is 15 and 30 nm for cylindrical and spherical particles, respectively [61,62]. Therefore, 10 to 30 nm diameter ENMs that have a spherical or similar shape appear to have the potential for more profound biological effects than either smaller or larger ENMs.

It is prudent to apply the continually improving understanding of the influence of the physico-chemical properties of ENMs on their effects and safety to the development of future ENMs, to enhance their benefit/risk ratio. Second generation (active) ENMs are being developed, such as targeted control-release systems for drugs. There is utility in the use of CNTs as drug delivery systems. Based on the studies of the role of CNT physico-chemical properties in biological effects it has been concluded that the use of low aspect ratio (length ≤ 1 μm), high purity (97-99%), low metal catalyst content CNTs minimizes cytotoxicity and provides apparent *in vivo *bio-compatibility [63]. Application of the continued understanding of the influence of physico-chemical properties on biological responses can similarly enhance the benefit/risk ratio of future ENMs, such as: application of the most predictive dose metric; the rate and nature of interacting proteins and effect of opsonization on uptake, translocation and effects; the influence of size, shape, charge, and surface reactivity on the extent and sites of translocation; and the duration of persistence of ENMs in organs and associated effects. Additionally, observations of workers exposed to ENMs can greatly add to this understanding, to increase confidence in the predicted effects of future ENMs.

#### a. The role of surface coating in ENM uptake and effects

ENMs are rapidly coated in biological milieu, primarily by proteins [62,64-66]. Due to high energetic adhesive forces close to the surface, ENMs can agglomerate and adsorb to the next available surface and other small molecules [67]. Extensive addition of polyethylene glycol (PEG) to the surface of SWCNTs has been shown to favor uptake into tumors compared to normal organs [68]. Similarly, addition of PEG to poly(di-lactic acid-co-malic acid) coated magnetic ENMs enhanced their uptake by macrophages [69]. Commercial providers and researchers often add a surface coating to inhibit ENM agglomeration and/or influence their uptake and cellular effects [70]. Cells that line the airways produce mucus. Pulmonary type II alveolar cells secrete surfactants (a mixture of 90% phospholipids and lung surfactant-specific proteins). Lung surfactants incorporate ENMs [71,72]. Mucus, which is secreted by goblet cells in the respiratory tract, eye, nasal cavity, stomach, and intestine, entraps ENMs [65]. All of these surface coatings on ENMs would be expected to affect their uptake and effects.

#### b. ENM uptake from the initial sites of exposure

To understand ENM-induced effects and their mechanisms of action, cells in culture and other *in vitro *systems have been utilized. However, these systems cannot model the complexities of the entire organism, including the limitation of uptake provided by such barriers as the skin and first-pass metabolism, opsonization, metabolism that may inactivate or activate a substrate, translocation to distal sites, activation of homeostatic defenses, or inflammatory processes that release cytokines and other factors that can act at distant sites from their release. Therefore, this review primarily cites examples of whole-animal studies to address ENM uptake and translocation.

##### i). Lungs

There has been much interest in the health effects of airborne particles, specifically PM_10 _(thoracic fraction), PM_2.5 _(respirable fraction), PM_1_, and ultrafine particles (PM_0.1_), which are ≤ 10, 2.5, 1 and 0.1 μm (100 nm), respectively. One- to 5-nm air-suspended ENMs that enter the lungs are not predicted to reach the alveoli; instead a high percentage is likely to deposit in the mucus-lined upper airways (tracheo-bronchial region) due to their strong diffusion properties. On the other hand ~45% of 10-nm, ~50% of 20-nm, and ~25% of 100-nm ENMs deposit in the alveoli [73]. Deposition is greater during exercise. Chronic obstructive pulmonary disease increases tracheo-broncheolar and decreases alveolar particle deposition [74,75].

##### ii) Nasal cavity

Uptake from the nasal cavity into the olfactory nerve, followed by retrograde axonal transport to the olfactory bulb and beyond, was shown in studies of the polio virus (30 nm) and colloidal silver-coated gold (50 nm) [76-78]. Uptake of ~35-nm ^13^C particles along the olfactory pathway to the olfactory bulb, and to a lesser extent into the cerebrum and cerebellum, was shown 1 to 7 days later [79]. Exposure to ~30 nm agglomerates of Mn by inhalation resulted in up to a 3.5-fold increase of Mn in the olfactory bulb, and lower (but significant) increases in 4 rat brain regions. The increase of Mn in brain regions other than the olfactory bulb may have resulted from translocation to the brain by route(s) other than via the olfactory nerve, such as through cerebrospinal fluid or across the blood-brain barrier [80]. The nasal cavity is the only site where the nervous system is exposed directly to the environment. This is an often overlooked potential route of uptake of small amounts of ENMs into the brain.

##### iii.) Dermal exposure

Skin is composed of 3 primary layers, the outermost epidermis (which contains the stratum corneum, stratum granulosum and stratum spinosum), dermis, and hypodermis. The hair follicle is an invagination of the stratum corneum, lined by a horny layer (acroinfundibulum). Dermal uptake routes are intercellular, intracellular, and follicular penetration. Uptake is primarily by diffusion. Materials that diffuse through the lipid-rich intercellular space of the stratum corneum typically have a low molecular weight (< 500 Da) and are lipophilic. Materials that penetrate the stratum corneum into the stratum granulosum can induce the resident keratinocytes to release pro-inflammatory cytokines. Materials that penetrate to the stratum spinosum, which contains Langerhans cells (dendritic cells of the immune system), can initiate an immunological response. This is mediated by the Langerhans cells, which can become antigen-presenting cells and can interact with T-cells. Once materials reach the stratum granulosum or stratum spinosum there is little barrier to absorption into the circulatory and lymphatic systems. Whereas dry powder ENMs pose a greater risk for inhalation exposure than those in liquids, liquid dispersed ENMs present a greater risk for dermal exposure.

Consumer materials most relevant to dermal exposure include quantum dots, titania, and zinc oxide in sunscreens, and silver as an anti-microbial agent in clothing and other products. Prolonged dermal application of microfine titania sunscreen suggested penetration into the epidermis and dermis [81]. However, subsequent studies did not verify penetration of titania from sunscreens into the epidermis or dermis of human, porcine or psoriatic skin [82-87], or find evidence of skin penetration of zinc oxide from sunscreen or positively- or negatively-charged iron-containing ENMs [88,89]. Nanoparticles with a dye penetrated deeper into hair follicles of massaged porcine skin *in vitro *and persisted longer in human skin *in vivo *than the dye in solution [82,90,91]. Thirty-nm carboxylated quantum dots applied to the skin of mice were localized in the folds and defects in the stratum corneum and hair follicles. A small amount penetrated as deep as the dermis. Ultraviolet radiation increased penetration, raising concern that these results might generalize to nanoscale sunscreens [92]. PEG-coated ~37 nm quantum dots accumulated in the lymphatic duct system after intra-dermal injection in mice. Cadmium, determined by ICP-MS, from cadmium-containing quantum dots was seen in liver, spleen, and heart; however, it is uncertain if this was from dissolved cadmium or translocation of the quantum dots because methods were not used to show the presence of quantum dots. The above results suggest topically-applied ENMs that penetrate to the dermis might enter the lymphatic system, and the ENMs or dissolved components distribute systemically [93]. To address these concerns ENMs intended for dermal application, such as titania, are often surface coated, e.g. with silica, alumina, or manganese. One goal of the surface treatments is to minimize toxicity by trapping the free radicals of reactive oxygen species (ROS) [94].

An *in vitro *study showed that mechanical stretching of human skin increased penetration of 500 and 1000 nm fluorescent dextran particles through the stratum corneum, with some distribution into the epidermis and dermis [95]. Similarly, mechanical flexing increased penetration of a 3.5 nm phenylalanine-based C_60 _amino acid ENM through porcine skin *in vitro *[96]. The contribution of skin flexing and immune system response was further addressed with three titania formulations applied to minipigs. There was some ENM penetration into epidermis and abdominal and neck dermis, but no elevation of titanium in lymph nodes or liver [97]. Topical exposure of mice to SWCNTs resulted in oxidative stress in the skin and skin thickening, demonstrating the potential for toxicity not revealed by *in vitro *studies of ENM skin penetration [98]. There are no reports of long-term studies with topical ENM exposure.

In the absence of organic solvents, the above suggests that topically applied ENMs do not penetrate normal skin. Not surprisingly, organic solvents (chloroform > cyclohexane > toluene) increased penetration of fullerene into skin that had the stratum corneum removed by tape stripping [99]. As the fullerenes were not detected in systemic circulation, there was no evidence of systemic absorption.

##### iv.) Oral exposure

Little is known about the bioavailability of ENMs from the buccal cavity or the sub-lingual site, or possible adverse effects from oral ingestion.

Particle absorption from the intestine results from diffusion though the mucus layer, initial contact with enterocytes or M (microfold or membranous specialized phagocytic enterocyte) cells, cellular trafficking, and post-translocation events [100]. Colloidal bismuth subcitrate particles (4.5 nm at neutral pH) rapidly penetrated the mucosa of dyspeptic humans, resulting in bismuth in the blood. Particles appeared to penetrate only in regions of gastric epithelial disruption [101]. Greater uptake of 50 to 60 nm polystyrene particles was seen through Peyer's patches and enterocytes in the villous region of the GI tract than in non-lymphoid tissue, although the latter has a much larger intestinal surface area [102,103]. Peyer's patches are one element of gut-associated lymphoid tissue, which consist of M cells and epithelial cells with a reduced number of goblet cells, resulting in lower mucin production [100,103]. It was estimated that ~7% of 50-nm and 4% of 100-nm polystyrene ENMs were absorbed [104]. Fifty-nm polystyrene ENMs fed to rats for 10 days by gavage showed 34% absorption, of which about 7% was in the liver, spleen, blood, and bone marrow; no ENMs were seen in heart or lung [104]. After oral administration of 50-nm fluorescence-labeled polystyrene ENMs, 18% of the dose appeared in the bile within 24 h and 9% was seen in the blood at 24 h; none was observed in urine [105]. The mechanism of GI uptake of 4, 10, 28 or 58 nm colloidal (maltodextran) gold ENMs from the drinking water of mice was shown to be penetration through gaps created by enterocytes that had died and were being extruded from the villus. Gold abundance in peripheral organs inversely correlated with particle size [106].

In summary, there appears to be significant absorption of some ENMs from the GI tract, with absorption inversely related to ENM size. The absorption site seems to be regions of compromised gastric epithelial integrity and low mucin content.

##### v.) Ocular and mucous membrane exposure

Ocular exposure might occur from ENMs that are airborne, intentionally placed near the eye (e.g., cosmetics), accidently splashed onto the eye, or by transfer from the hands during rubbing of the eyes, which was shown to occur in 37% of 124 adults every hour [107]. This route of exposure could result in ENM uptake through the cornea into the eye or drainage from the eye socket into the nasal cavity through the nasolacrimal duct. Other than a study that found uptake of a polymer ENM into conjunctival and corneal cells, this route has been largely ignored in research studies of ENM exposure [108].

### B. The effects of ENM exposure on target organs and those distal to the site of uptake

Public concerns about ENMs and health may arise with reports of some effect(s) in a laboratory study or their presence in human tissue (or another organism). Any report must be interpreted carefully before concluding ENMs are risky for one's health. To start with, risk is defined as a joint function of a chemical's ability to produce an adverse effect and the likelihood (or level) of exposure to that chemical. In a sense, this is simply a restatement of the principle of dose-response; for all chemicals there must be a sufficient dose for a response to occur. Additionally, advances in analytical chemistry have led to highly sensitive techniques that can detect chemicals at remarkably low levels (e.g., in parts per billion or parts per trillion). The detectable level may be far lower than any dose shown to produce an adverse effect. Further, a single finding in the literature may garner public attention, and it may be statistically significant, but its scientific importance remains uncertain until it is replicated, preferably in another laboratory. In this regard, a follow-up study may be warranted to characterize the relevant parameters of dose, duration, and route of exposure, as outlined in the Risk Assessment/Risk Management framework.

The above discussion reflects many of the issues that have gained prominence in the fields of risk perception and risk communication (see for example [109,110]), neither of which were dealt with by the NRC in their landmark publication.

The knowledge of ultrafine-particle health effects has been applied to ENMs. However, the toxicity from ultrafine materials and ENMs is not always the same [111]. Similarly, the effects produced by ENM components do not reliably predict ENM effects. For example, toxicity was greater from cadmium-containing quantum dots than the free cadmium ion [112]. Some metal and metal oxide ENMs are quite soluble (e.g., ZnO), releasing metal ions that have been shown to produce many of the effects seen from ENM exposure [113,114]. Therefore, one cannot always predict ENM toxicity from the known effects of the bulk or solution ENM components.

#### 1. ENM exposure effects in the lung

Studies of ENM inhalation and intratracheal instillation as well as with lung-derived cells in culture have increased concern about potential adverse health effects of ENMs. An early 2-year inhalation study of Degussa P-25 (a ~3:1 mixture of ~85-nm anatase and 25-nm rutile titania) resulted in lung tumors in rats [115]. SWCNTs containing residual catalytic metals produced greater pulmonary toxicity, including epithelioid granulomas and some interstitial inflammation, than ultrafine carbon black or quartz. These effects extended into the alveolar septa [116]. A review of eleven studies of carbon nanotube introduction to the lungs of mice, rats, and guinea pigs revealed most found granuloma, inflammation, and fibrosis [117]. MWCNTs produced greater acute lung and systemic effects and were twice as likely to activate the immune system as SWCNTs, suggesting the former have greater toxic potential [118]. Further adding to the concern of ENM-induced adverse health effects are reports that inhaled CNTs potentiate airway fibrosis in a murine model of asthma [119], and that exposure of a cell line derived from normal human bronchial epithelial (BEAS-2B) cells to SWCNTs and graphite nanofibers produced genotoxicity and decreased cell viability [120]. However, a point of contention is that the lung response to intratracheal and inhaled MWCNTs differed among studies. This may have been due to different sizes and distributions of MWCNT agglomerations. These differences create uncertainties regarding the utility of some routes of pulmonary ENM exposure used in laboratory studies to predict potential toxicity in humans [121].

Studies exposing lung-derived cells in culture to ENMs have demonstrated similar effects. Carbon-based ENMs produced pro-inflammatory, oxidative-stress, and genotoxic effects [122,123].

Several groups have studied the effects of CNT introduction into the peritoneal cavity. As there is CNT translocation from the lung to other sites (see **II, D**. **Clearance of ENMs, their translocation to distal sites, and persistence)**, and the internal surfaces of the peritoneal and pleural cavities are lined with a mesothelial cell layer, responses in the peritoneal cavity appear to be relevant to the pleural cavity. Single ip injection of high-aspect-ratio MWCNTs (~100 nm diameter and 2000 nm long) produced inflammation, granulomatous lesions on the surface of the diaphragm, and mesothelioma that were qualitatively and quantitatively similar to those caused by crocidolite asbestos, also a high-aspect-ratio fiber [124]. These effects correlated positively with the MWCNT aspect ratio [125,126].

Toxicity has also been seen from pulmonary introduction of metal and metal oxide ENMs. Ten and 20 nm anatase titania induced in BEAS-2B cells oxidative DNA damage, lipid peroxidation, increased H_2_O_2 _and nitric oxide production, decreased cell growth, and increased micronuclei formation (indicating genetic toxicity) [52]. Exposure of BEAS-2B cells to 15- to 45-nm ceria or 21-nm titania resulted in an increase of ROS, increased expression of inflammation-related genes, induction of oxidative stress-related genes, induction of the apoptotic process, decreased glutathione, and cell death [127,128]. Twenty-nm ceria increased ROS generation, lipid peroxidation, and cell membrane leakage, and decreased glutathione α-tocopherol (vitamin E) and cell viability in a human bronchoalveolar carcinoma-derived cell line (A549) [129]. Various metal oxides differentially inhibited cell proliferation and viability, increased oxidative stress, and altered membrane permeability of human lung epithelial cells [130].

#### 2. ENM exposure effects seen in the brain

Murine microglial cells were exposed to a commercial 70%:30% anatase:rutile titania (primary crystalline size 30 nm; 800 to 2400 nm agglomerations in test medium). They displayed extracellular release of H_2_O_2 _and the superoxide radical and hyper-polarization of mitochondrial membrane potential [131]. Intravenous ceria administration to rats altered brain oxidative stress indicators and anti-oxidant enzymes [23,132]. These results demonstrate the ability of metal oxide ENMs to produce neurotoxicity.

#### 3. ENM exposure effects seen in the skin

Potential toxicity from dermal exposure was demonstrated with silver ENMs, that decreased human epidermal keratinocyte viability [133]. These results demonstrate the ability of metal oxide ENMs to also produce dermatotoxicity.

#### 4. Summary of the effects of ENM exposure on target organs and those distal to the site of uptake

Common findings of many studies are induction of inflammatory processes and oxidative stress. However, correspondence between responses of cells in culture and *in vivo *models is often low [24,43]. In light of the pressure to minimize whole animal (e.g., rodent) research, further development of cell-based or *in vitro *models of the whole organism is expected. Additionally, there has been considerable use of alternative model organisms e.g., *C. elegans*, which has a genome with considerable homology with vertebrate genomes and is often used in ecotoxicological studies, and zebrafish which are often used in developmental biology and genetic studies [134-136].

### C. Dose-response assessment

Exposure in experimental studies is typically expressed as dose, usually on a mass/subject body weight basis, or as concentration. Dose or concentration may not be the best metric to predict ENM effects [42,53,137]. Neutrophil influx following instillation of dusts of various nanosized particles to rats suggested it may be more relevant to describe the dose in terms of surface area than mass [138]. The pro-inflammatory effects of *in vitro *and *in vivo *nanoscale titania and carbon black best correlated when dose was normalized to surface area [122]. Secretion of inflammatory proteins and induction of toxicity in macrophages correlated best with the surface area of silica ENM [139]. Analysis of *in vitro *reactive oxygen species generation in response to different sized titania ENMs could be described by a single S-shaped concentration-response curve when the results were normalized to total surface area, further suggesting this may be a better dose metric than concentration [53]. Similarly, using surface area as the metric, good correlations were seen between *in vivo *(PMN number after intratracheal ENM instillation) and *in vitro *cell-free assays [42].

Nonetheless, most studies of ENMs have expressed exposure based on dose or concentration. The relatively small amount of literature has generally shown dose- or concentration-response relationships, as is usually the case for toxicity endpoints. Ceria ENM uptake into human lung fibroblasts was concentration-dependent for several sizes, consistent with diffusion-mediated uptake [58]. Positive, dose-dependent correlations were seen in blood, brain, liver, and spleen following iv ceria infusion in rats, measured by elemental analysis as cerium [23], as well as brain titanium after ip titania injection [140], and lung cobalt after inhalation of cobalt-containing MWCNTs [141]. Concentration-dependent inhibition of RAW 264.7 (murine) macrophage cell proliferation was seen following *in vitro *SWCNT exposure, as was lipopolysaccharide-induced COX-2 expression, up to 20 μg/ml [142]. Intratracheal instillation of MWCNTs (average length ~6 μm) or ground MWCNTs (average length ~0.7 μm) produced dose-dependent increases in LDH activity and total protein, but no dose-dependent effect on the number of neutrophils or eosinophils, or TNF-α, in rat lung bronchoalveolar lavage fluid [143]. Activated Kupffer cell count increased with iv ceria dose; the increase in hippocampal 4-hydroxy-2-*trans*-nonenal and decrease in cerebellar protein carbonyls (indicators of oxidative stress) were dose-dependent up to a maximum that did not increase further at the highest dose [23].

Some studies demonstrating adverse effects of CNT introduction to the lung have been criticized for using doses or concentrations that far exceeded anticipated human exposure [144]. Most studies assessing potential adverse effects of ENMs have utilized a single exposure. Both of these features make extrapolation of results to prolonged or episodic (periodic) human exposure difficult. However, the study of acute high doses/concentrations to probe potential effects is a standard approach in toxicology and experimental pathology for initially surveying adverse effects (i.e., hazard identification). When adverse effects are seen following some reasonable (e.g., sublethal) dose, subsequent studies must define exposures that do, and do not, result in adverse effects.

### D. The clearance of ENMs, their translocation to distal sites, and persistence

As with the above studies that inform about uptake, the clearance and translocation of ENMs from the initial site of exposure to distal sites is best understood from whole-animal studies.

The solutes of dissolved particles in the lung can transfer to blood and lymphatic circulation. Some ENMs in the airway wall that slowly dissolve or are insoluble will be cleared within a few days from the lung by cough or the mucociliary escalator. Slowly dissolving and insoluble ENMs that reach the alveoli may be taken up by macrophages. Macrophage-mediated phagocytosis is the main mechanism for clearing foreign material from the deep lungs (alveoli) and from other organs. Macrophages are ~20 μm in diameter and able to phagocytose materials up to 15 μm in length. They engulf the particle in a vacuole (phagosome) containing enzymes and oxidizing moieties that catabolize it. Particles resistant to catabolism may remain inside the macrophage. After the death of the macrophage the material may be engulfed by another cell. Therefore, it may take a long time for insoluble material to be cleared from the body. The elimination half-live of insoluble inert particles from the lung can be years [145]. This raises the question of the ultimate fate of "poorly digestible" ENMs that are engulfed by macrophages in the lung, liver (Kupffer cells), brain (microglia), and other organs.

Some ENMs, e.g., those that have a high aspect ratio, are not effectively cleared by macrophages. Alveolar macrophages that cannot digest high-aspect-ratio CNTs (termed "frustrated phagocytosis") can produce a prolonged release of inflammatory mediators, cytokines, chemokines, and ROS [146]. This can result in sustained inflammation and eventually fibrotic changes. Studies have demonstrated MWCNT-induced pulmonary inflammation and fibrosis, similar to that produced by chrysotile asbestos and to a greater extent than that produced by ultrafine carbon black or SWCNTs [117]. Greater toxicity from a high-aspect-ratio metal oxide (titania) ENM has also been shown in cells in culture and *in vivo *[147]. Studies such as these have raised questions (and concern) about the long-term adverse effects of ENM exposure.

Translocation of ENMs from the lung has been shown. After MWCNT inhalation or aspiration they were observed in subpleural tissue, the site of mesotheliomas, where they caused fibrosis [148,149]. Once ENMs enter the circulatory system across the 0.5-μm thick membrane separating the alveoli from blood, the sites of reticuloendothelial system function (including the lymph nodes, spleen, Kupffer cells, and microglia) clear most ENMs. Thirty to 40 nm insoluble ^13^C particles translocated, primarily to the liver, following inhalation exposure [150]. Similarly 15 and 80 nm ^192^iridium particles translocated from lung to liver, spleen, heart, and brain. The extent of translocation was < 0.2%, and greater with the smaller ENMs [151].

ENMs have also been shown to translocate following injection. Indirect evidence was shown of fullerene distribution into, and adverse effects in, the fetus 18 h after its injection into the peritoneal cavity of pregnant mice on day 10 of gestation [152]. Following subcutaneous injection of commercial 25 to 70 nm titania particles into pregnant mice 3, 7, 10, and 14 days post coitum, aggregates of 100 to 200 nm titania were seen in the testes of offspring at 4 days and 6 weeks post-partum and in brain at 6 weeks post-partum. Abnormal testicular morphology and evidence of apoptosis in the brain indicated fetal titania exposure had adverse effects on development. The authors attribute these effects to ENM translocation across the placenta [153]. ENM excretion into milk and oral absorption post-partum might contribute to ENM presence in the offspring, but we are unaware of any studies assessing ENM translocation into milk. Non-protein bound substances generally enter milk by diffusion, and reach an equilibrium between milk and blood based on their pKa and the pH difference between blood and milk, described by the Henderson-Hasselbalch equation. Given the size of most ENMs, it is unlikely they would diffuse across the mammary epithelium. Within 40 weeks after a single intrascrotal injection of MWCNTs most rats died or were moribund with intraperitoneal disseminated mesothelioma, which were invasive to adjacent tissue, including the pleura. Fibrous MWCNT particles were seen in the liver and mesenteric lymph nodes, suggesting peritoneal effects might have been due to MWCNT translocation [154].

The distribution of carbon-, metal- and metal oxide-based ENMs after translocation from the lung, skin or intestine is similar to that seen after their iv administration. They generally appear as agglomerates in the liver and spleen [23,93,132,151,155-158]. The ENMs are usually in the cytoplasm, with little indication that they enter the nucleus [132,134,158-160].

Due to their small size ENMs may gain access to regions of the body that are normally protected from xenobiotics (sanctuaries), such as the brain. This feature has suggested their potential application for drug delivery to the brain, which is being extensively pursued [161-164], but at the same time it raises concern about central nervous system distribution of ENMs when exposure is not intended. Studies have generally found << 1% of the administered dose of ceria and iridium ENMs translocate to the brain after inhalation exposure or iv injection [23,132,151]. Anionic polymer ENMs entered the brain more readily than neutral or cationic ones. Both anionic and cationic ENMs altered blood-brain barrier integrity [165].

The persistence of ENMs may be a major factor contributing to their effects. Many ENMs are designed to be mechanically strong and resist degradation [22]. Referring to nanoscale fiber-like structures, it has been stated: "The slower [they] are cleared (high bio-persistence) the higher is the probability of an adverse response" [166]. The analogy of high-aspect-ratio ENMs to asbestos is one of the contributors to this concern. The prolonged physical presence of ENMs, that are not metabolized or cleared by macrophages or other defense mechanisms, appears to elicit ongoing cell responses. The majority of CNTs are assumed to be biopersistent. For example, two months after the intratracheal instillation of 0.5, 2 or 5 mg of ~0.7 μm and ~6 μm MWCNTs, 40 and 80% of the lowest dose remained in the lungs of rats, suggesting adequate persistence to cause adverse effects that are summarized in **II, B, 1 ENM exposure effects in the lung **[143]. Following oral administration, 50-nm non-ionic polystyrene ENMs were seen in mesenteric lymphatic tissues, liver, and spleen 10 days later [167]. Following iv administration, carboxylated-MWCNTs were cleared from circulation and translocated to lung and liver; by day 28 they were cleared from the liver, but not from the lung [168]. No significant decrease of the amount (mass) of cerium was seen in the liver or spleen of rats up to 30 days after iv administration of 5 or 30 nm ceria. Hepatic granuloma and giant cells containing agglomerates in the cytoplasm of the red pulp and thickened arterioles in white pulp were seen in the spleen (unpublished data, R. Yokel) [159,169].

In summary, the persistence of ENMs in tissue raises justifiable concerns about their potential to cause long-term or delayed toxicity.

### E. The physico-chemical properties of ENMs that impact their hazard - The role of surface coating in ENM effects

Many surface coatings have been investigated in order to develop ENMs as carriers for drug delivery. Surface modifications can prolong ENM circulation in blood, enhance uptake at a target site, affect translocation, and alter excretion. When ENMs enter a biological milieu they rapidly become surface coated with substances such as fulvic and humic acids and proteins, all of which can alter their effects [142,170,171]. When 3.5, 20, and 40 nm gold and DeGussa P-25 titania ENMs were incubated with human plasma, proteins appeared to form a monolayer on the ENMs. The abundance of plasma proteins on gold approximated their abundance in plasma, whereas some proteins were highly enriched on titania [172]. Metal oxide and carbon-based ENMs rapidly adsorb proteins [66], resulting in changes in their zeta potential (electrical potential at the ENM surface) and toxicity [142,171]. For circulating ENMs, the surface coating is extremely important, because this is what contacts cells [173].

Although it is understood that ENMs will be surface coated with proteins, lipids or other materials, which may or may not persist on the ENM surface when they enter cells, little is known about the surface associated molecules on ENMs within cells. It is likely, however, that surface coatings profoundly influence ENM effects within cells. Although surface functional groups are known to modify ENM physico-chemical and biological effects, there is little information on the influence of functional groups on health effects. This further complicates the prediction of ENM toxicity in humans from *in vitro*, and perhaps *in vivo*, studies.

### F. The effects of ENMs at distal sites

Reported systemic effects of pulmonary-originating CNTs include acute mitochondrial DNA damage, atherosclerosis, distressed aortic mitochondrial homeostasis, accelerated atherogenesis, increased serum inflammatory proteins, blood coagulation, hepatotoxicity, eosinophil activation (suggesting an allergic response), release of IL-6 (the main inducer of the acute phase inflammatory response), and an increase of plasminogen activator inhibitor-1 (a pro-coagulant acute phase protein) [118]. Elevation of the serum analyte ALT was reported up to 3 months after intratracheal MWCNT instillation, suggesting ENM-induced hepatotoxicity [174]. The translocation of ENMs and their release of cytokines and other factors could potentially affect all organ systems, including the brain. For example, daily ip injection of titania for 14 days resulted in a dose-dependent increase of titanium and oxidative stress and a decrease of anti-oxidative enzymes in the brain of rats [140].

## III. Hazard Assessment from Fire and Explosion of ENMs

Some ENMs have very high reactivity for catalytic reactions, thus raising the possibility of fire and/or explosion. As particle size decreases and surface area increases, the ease of ignition and the likelihood of a dust explosion increase. The latter may create a second hazard due to increased ENM release. There are no reports that ENMs have been used intentionally, e.g. by terrorists, or unintentionally to cause fires, explosions, or an airborne obscurant effect.

## IV. Exposure Assessment

Another key element of the Risk Assessment/Risk Management framework is exposure assessment, which includes the most likely routes of ENM exposure. Not much is known about the extent of occupational exposure to ENMs. There are ~20 published studies [51]. "In the absence of solid exposure data, no solid risk evaluation can be conducted" [175]. There is obvious value in conducting exposure assessments in the workplace to identify the routes, extent, and frequency of ENM exposure. In assessing worker exposure, the traditional industrial hygiene sampling method of collecting samples in the breathing zone of the worker (personal sampling) is preferred over area sampling. Only a few of the studies cited [51] conducted breathing zone measurements. On the other hand, area samples (e.g., size-fractionated aerosol samples) and real-time (direct-reading) exposure measurements are useful for evaluating engineering controls, and their efficacy, and work practices.

When monitoring potential workplace exposure to ENMs it is critical that background nanoscale particle measurements be conducted before their production, processing, or handling in order to obtain baseline data. Nanosize particles frequently come from non-ENM sources, such as ultrafines from internal combustion engines and welding [176,177].

An early study of SWCNT release during its handling in the workplace showed very low airborne concentrations of agglomerated material [178]. The rapid agglomeration of ENMs in air has been repeatedly shown [55,178,179]. Airborne ENMs associate with other airborne materials when present, or self-associate in their absence. Once formed there was little decrease in the resultant airborne agglomerations for up to 4 h [55]. An on-site monitoring study of carbon nanofibers (CNFs) in a university-based research laboratory showed an increase of > 500-nm particles in air during weighing and mixing (total carbon levels in inhalable dust samples of 64 and 93 μg/m^3^, respectively). Handling the bulk partially-dry product on the lab bench generated 221 μg/m^3^, and wet-saw cutting (which sprays water on the object being cut to lessen dusts) of a CNF composite released > 400-nm particles (1094 μg/m^3^). Office background was 15 to 19 μg/m^3^. Surface samples had up to 30-fold more total carbon than the office floor [180]. Another study showed that wet cutting of a hybrid CNT in an epoxy resin or in a woven alumina fiber cloth using a cutting wheel with water to flush dust particles produced no significant increase of airborne 5- to 1000-nm particles in the operator breathing zone, whereas dry machining did [181]. Production of a nanocomposite containing alumina in a polymer by a twin-screw extrusion process caused release of 5- to 20-nm and 50- to 200-nm alumina in the worker's breathing zone [182]. Covering the top of the feeding throat and the open mouth of the particle feeder, thorough cleaning by washing the floor, and water-based removal of residual dust on all equipment significantly decreased airborne particles [182,183]. These results suggest that some engineering controls may be appropriate to safely remove some airborne ENMs, including maintaining the room at negative pressure relative to the outside, avoiding the handling of dry ENMs, adequate ventilation, and containment of the ENM material during its use.

NIOSH researchers developed a Nanoparticle Emission Assessment Technique (NEAT) for use in the workplace [56]. They used the technique to determine particle number concentrations using two hand-held, direct-reading, particle number concentration-measuring instruments, a condensation and an optical particle counter, to survey 12 sites working with ENMs. This was complemented by collection of particles on filters and transmission electron microscopic visualization. The results demonstrated the utility of NEAT and, in some cases, the source of ENM release and efficacy of engineering controls [179]. Engineering controls are discussed in more detail below.

There are numerous reports of adverse lung effects, and some reports of human deaths, from nanosized polymer fumes[26]. Two deaths were reported among seven 18- to 47-year-old female workers exposed to polyacrylate nanoparticles for 5 to 13 months. Cotton gauze masks were the only PPE used, and were used only occasionally. The workplace had one door, no windows, and no exhaust ventilation for the prior 5 months [184]. Workers presented with dyspnea on exertion, pericardial and pleural effusions, and rash with intense itching. Spirometry showed that all suffered from small airway injury and restrictive ventilatory function; three had severe lung damage. Non-specific pulmonary inflammation, fibrosis, and foreign-body granulomas of the pleura were seen. Fibrous-coated nanoparticles (~30 nm) were observed in the chest fluid and lodged in the cytoplasm, nuclei, and other cytoplasmic organelles of pulmonary epithelial and mesothelial cells. Two workers died of respiratory failure. Although presented as the first report of clinical toxicity in humans associated with long-term ENM exposure, many experts have expressed uncertainty that ENMs contributed to these outcomes [22,185,186]. Given the poor environmental conditions of the workplace and lack of effective PPE use, these outcomes could probably have been prevented.

## V. Risk Characterization

The giant insurance firm Lloyd's of London conducted a risk assessment and concluded "Our exposure to nanotechnology must therefore be considered and examined very carefully" [http://www.nanolawreport.com/2007/12/articles/review-lloyds-new-nano-insurance-report/]. Japan's Ministry of Health, Labour and Welfare funded studies starting in 2005 to establish health risk assessment methodology of manufactured nanomaterials. It was recently concluded that studies of metals and SWCNTs from Japan are not yet sufficient to evaluate ENM risk [187]. However, a new study incorporated a physiologically-based lung model and data of particle sizes of airborne titania ENM during manufacturing to estimate anatase and rutile titania ENM burdens and adverse effects in lung cells. The authors concluded that workers exposed to relatively high airborne 10- to 30-nm anatase titania are unlikely to have substantial risk for lung inflammatory responses, but are at risk for cytotoxicity [188]. Risk characterization and assessment and gap analysis case studies were conducted with fullerenes, CNTs, silver as a example of a metal, and titania as an example of a metal oxide ENM [189]. Numerous additional data gaps were identified for each.

## VI. Risk Management

There are no existing regulations or standards for ENMs within the three jurisdictions that have the largest nanotechnology funding, the U.S., EU and Japan [190]. In the U.S. OSHA would set standards for occupational exposure to ENMs. Three types of standards are relevant for ENMs under the Occupational Safety and Health Act [191]. 1) Substance-specific standards, for which there are none for ENMs. 2) General respiratory protection standards, under which inhalable ENMs would be considered particulates not otherwise regulated, e.g. "nuisance dust", with a 5 mg/m^3 ^time-weighted average air exposure limit, determined by breathing-zone air samples. The respiratory protection standard requires employers provide workers with NIOSH-certified respirators or other PPE when engineering controls are not adequate to protect health. 3) The hazard communication standard states producers and importers of chemicals must develop Material Safety Data Sheets [191]. The U.S. EPA, using the legislative authority of the Toxic Substances Control Act has taken steps to limit the use and exposure to ENMs, including CNTs. EPA has required the use of PPE and limitiation on ENM use and environmental exposures [22]. NIOSH prepared a draft Current Intelligence Bulletin: "Occupational Exposure to Carbon Nanotubes and Nanofibers" (http://www.cdc.gov/niosh/docket/review/docket161A/pdfs/carbonNanotubeCIB_PublicReviewOfDraft.pdf). NIOSH recommends an 8-hour time-weighted average exposure limit of 7 μg carbon nanotubes and nanofibers/m^3 ^air, and that employers minimize exposure to these materials. Suggested implementation includes many of the primary prevention measures discussed in this review and an occupational health surveillance program of exposure and medical monitoring. Given the 7 μg/m^3 ^level is below total airborne carbon in non-CNT-production settings (offices) [180], the ubiquitous presence of CNTs which is probably due to hydrocarbon combustion [192], and the necessity to differentiate CNTs from other carbon sources to estimate airborne nanotube and nanofiber concentration, assuring their airborne level of < 7 μg/m^3 ^may be difficult.

**Figure 4:**
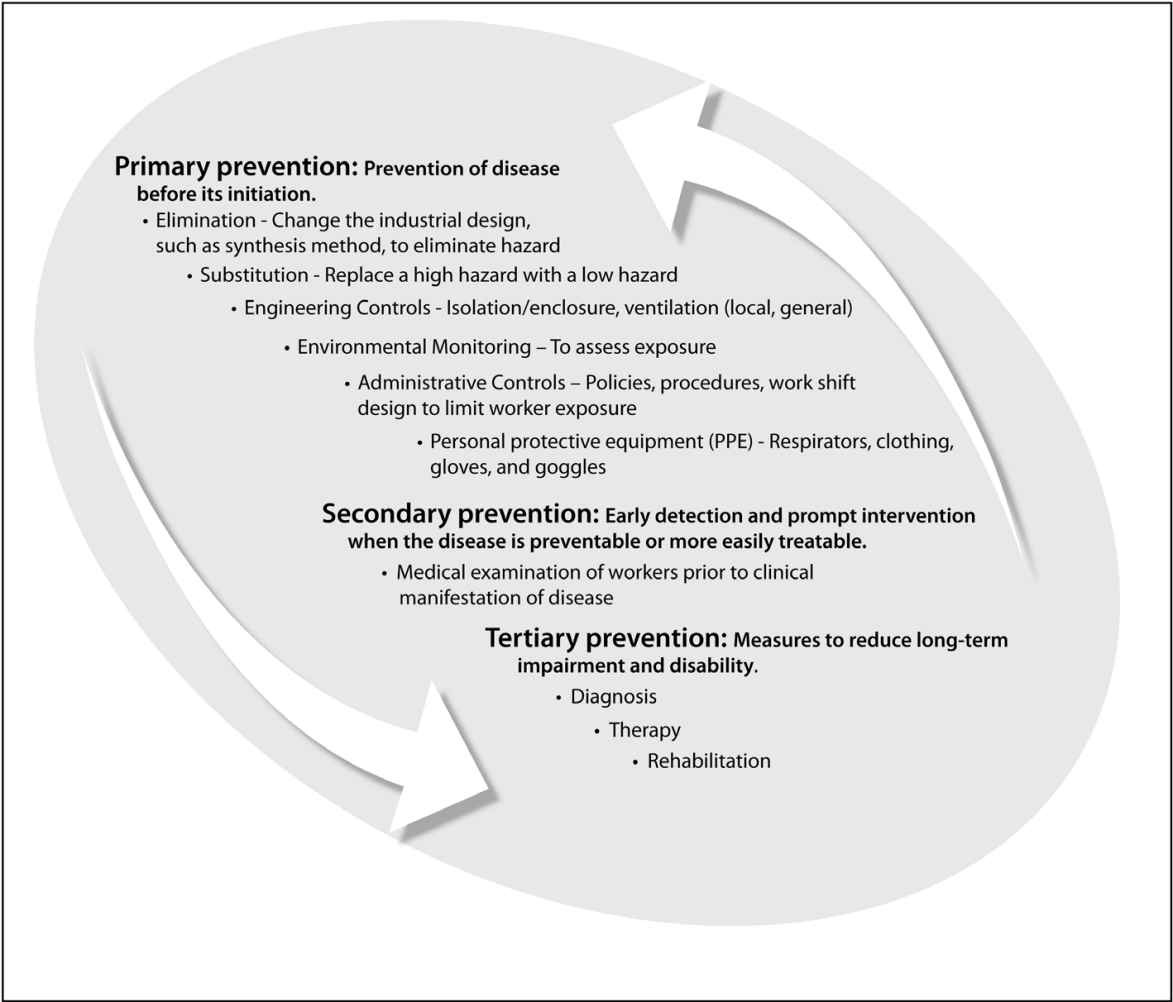
**Elements of occupational health protection**. The continuum of prevention and the heirarchy of exposure control (left arrow) and occupational health surveillance (right arrow). Adapted from [222] and [194].

The goal in managing the potential risks from ENMs is to minimize exposure. In the absence of specific information on ENMs, the extensive scientific literature on airborne, respirable aerosols and fibers has been used to develop interim guidance for working safely with ENMs [193] [http://ehs.mit.edu/site/content/nanomaterials-toxicity] [http://www.astm.org/Standards/E2535.htm].The general approach to minimizing exposure is shown in Figure 4, with the preferred followed by less desireable controls shown by the downward pointing arrow. Occupational health surveillance, which includes hazard and medical surveillance, is the process whereby information from any of these activities is collected and used to support or modify what is done at a higher step in the hierachy, as shown by the upward pointing arrow [194]. Those steps in the hierachy that have been investigated for ENMs are further discussed below.

### A. Engineering controls

ENM exposure can be reduced through the use of engineering controls, such as process changes, material containment, and enclosures operating at negative pressure compared to the worker's breathing zone; worker isolation; separated rooms; the use of robots; and local exhaust ventilation (LEV). Given the lack of occupational exposure standards to provide guidance, the most prudent approach is to minimize exposure. A survey found that engineering controls in Switzerland were more commonly used in the production of powder than liquid ENMs. For the former, the use of PPE (masks, gloves, safety glasses, or full protective suits) were the norm, although used by only ~16, 19, 19, and 8% of the workers, respectively [195]. This low use of PPE is thought to reflect the early stage of development of the ENM industry. It is anticipated that as this industry matures and knowledge is gained, control will more commonly include superior methods in the hierarchy of exposure control [196]. An international survey of ENM industry managers conducted in 2009-2010 by the University of California Center for Environmental Implications of Nanotechnology that focused on industry controls of ENM exposure, use of PPE, environmental risks, and perceptions revealed that 46% of the companies had a nano-specific environmental health and safety program, compared to 58% in a 2006 survey [197]. Some companies (a minority) were using inappropriate occupational environmental clean-up methods, such as sweeping and compressed air [198]. These results suggest more widespread adoption of nano-specific environmental health and safety programs and the use of PPE in the absence of superior controls are appropriate.

#### 1. Process containment

Process/source enclosure (i.e., isolating the ENM from the worker) can be aided by glove boxes, chemical fume hoods, biological safety cabinets (BSC), or an externally-vented LEV system. However, one should also consider that these methods can release ENMs into the environment, potentially creating environmental pollution and loss of costly material.

ENM handling is often conducted in fume hoods. Field sampling conducted to determine fume hood, work zone, and background concentrations of PM_2.5 _(< 2.5 μm) particles during production of fullerenes and other carbon-containing ENMs showed handling produced aerosolization of 5 to 100 nm particles, which were contained by the fume hood [199]. Monitoring aerosolized particles during chemical vapor deposition (CVD) SWCNT synthesis and aerosol-assisted CVD MWCNT synthesis in a fume hood showed significant release at the source, but not outside of the hood, suggesting fume hood use did not create fugitive airborne emissions and was necessary to protect workers [144]. These authors also determined the release of dry powder alumina (27 to 56 nm primary particle size, 200 nm agglomerates) and 60 nm silver ENM into the researcher's breathing zone and laboratory environment when poured or transferred in 3 fume hoods; 1) a conventional hood that has a constant air flow with velocity inversely related to sash height, 2) a by-pass hood which attempts to maintain a constant velocity by use of a by-pass grill above the hood which becomes uncovered, allowing more air flow through it rather than the hood face as the sash is lowered, and 3) a constant velocity (variable air volume) hood that uses a motor to vary fan speed as the sash is moved. The results showed significant release of ENMs into the researcher's breathing zone and laboratory environment and identified the variables affecting release. These included hood face velocities < 80 ft/min (< 0.4 m/s) (due to room air currents and operator movements) and > 120 ft/min (> 0.6 m/s) (due to turbulence within the hood). The constant velocity hood performed better than the by-pass hood, which in turn performed better than the conventional hood [200]. Tests were also conducted with alumina nanoparticles (primary particle size 27 to 56 nm; present as dry bulk material ~200 nm) to compare particles in the breathing zone during transfer and pouring in constant flow, constant velocity and air-curtain hoods. The newly developed air-curtain hood is evidently not commercially available. The results showed much lower levels with the air curtain hood [201]. Sash height, which affected hood face velocity, affected ENM release. Rapid removal of the worker's arm from a BSC also withdrew ENMs, releasing them outside the cabinet. Worker motion and body size affected ENM release from a traditional, but not the air-curtain, hood. The authors found that ENM handling in traditional fume hoods with a face velocity of 0.4 to 0.5 m/s (~80 to 100 ft/min) and careful motions minimized fugitive ENM emission. In 2008 the Center for High Rate Manufacturing recommended locating equipment at least 6 inches (15 cm) behind the sash, minimizing hood clutter, and avoiding rapid or violent motions while working in the hood [202]. In a study conducted in an industrial setting, use of an exhaust hood during procedures that are more likely to release ENMs (their production, handling, measurement, and reactor cleanout) resulted in no significant increase of ENMs in the workplace [203]. These studies show that significant reduction of worker exposure to ENMs can be achieved using available fume hoods and consideration of worker activities within these hoods.

Labconco Corporation has marketed a modified Class I BSC for handling nanoparticles [http://www.labconco.com/_scripts/editc20.asp?CatID=82]. It has an all stainless steel interior for ease of cleaning, perforated rear baffle to reduce turbulence, and a replaceable HEPA filter. It is available with a built-in ionizer to attract particles to the interior surface of the hood, and an external exhaust for volatiles.

#### 2. Local exhaust ventilation (LEV)

Air-displacement ventilation in an industrial setting was accomplished by introduction of supply air that entered at low velocity at the floor level and was cooler than room air. As the air rose it became warmer and was exhausted close to the ceiling. This provided efficient clearing of ENMs from the breathing zone [204].

A well-designed exhaust ventilation system with a HEPA filter should effectively capture airborne nanoparticles. A "down flow" booth, "elephant trunk", or fume hood may not provide sufficient protection because they may cause turbulence, spinning the ENM out of the airflow [201].

The effectiveness of engineering controls in ENM production and research facilities has been demonstrated in a few cases. Prior to use of engineering-control measures, total airborne mass concentrations of MWCNTs, measured by area sampling, were 0.21 to 0.43 mg/m^3 ^in a laboratory research facility where the powders were blended to formulate composites. After enclosing and ventilating the blending equipment and re-locating another piece of equipment that produced considerable vibration, the concentration decreased to below the limit of detection [205]. In another study, the effectiveness of LEV was assessed during clean-out of slag and waste, which used brushes and scrapers, of reactors that produced 15 to 50 nm diameter ENMs. A portable LEV unit was used that had been shown to reduce welding fume exposure [206]. The reduction in release of 300- to 10,000-nm Ag, Co, and Mn particles during cleanout of reactors used to make nanoscale metal catalytic materials was 75, 94, and 96%, respectively [207].

During the manual sanding of epoxy test samples reinforced with MWCNTs, an order of magnitude more particles, which were predominantly > 300 nm, was observed in a worker's breathing zone when the work was conducted in a custom fume hood rather than on a work table with no LEV. The poor performance of the custom fume hood may have been due to the lack of a front sash and rear baffles, and to low face velocity (0.39 m/sec). Respirable particles were an order of magnitude lower when the work was conducted in a BSC than on a work table [208]. These results illustrate the importance of good exhaust hood design as well as the worker protection provided by a BSC.

### B. Administrative controls

When engineering controls are not feasible for reducing exposure, administrative controls should be implemented. These are policies and procedures aimed at limiting worker exposure to a hazard [209]. These could include a nanoscale material hygiene plan; preparation, training in, and monitoring use of standard operating procedures; reduction of exposure time; modification of work practices; and good workplace and housekeeping practices. For example, one laboratory was thoroughly cleaned after high air concentrations of nanoscale materials were measured in a facility engaged in the commercial compounding of nanocomposites [183]. A large decrease of airborne 30 to 100 nm particles resulted. Subsequent routine maintenance kept the particles below those originally observed, leading the authors to conclude that this administrative control was beneficial in reducing potential exposure. Biological monitoring and medical examination, a component of secondary prevention (Figure 4), is another administrative control [209]. It is discussed below.

### C. Personal protective equipment

The last line of defense in the hierarchy of exposure control is the use of PPE, such as respirators, protective clothing, and gloves.

#### 1. Respirators

Major types of respiratory protection include dust masks, filtering facepiece respirators, chemical cartridge/gas mask respirators, and powered air-purifying respirators. Examples can be seen at OSHA's Respiratory Protection Standard site [http://web.utk.edu/~ehss/pdf/rpp.pdf].

NIOSH classifies filter efficiency levels as Type 95, 99, and 100, which are 95, 99, and 99.7% efficient, respectively. The filter's resistance to oil is designated as N, R, and P; N (not resistant to oil), R (resistant to oil), and P (oil proof). Some industrial oils can remove electrostatic charges from filter media, reducing filter efficiency. Efficiency of N filters is determined using 300-nm median aerodynamic, charge neutralized, NaCl particles at a flow rate of 85 l/min; R and P filters are tested with dioctyl phthalate oil. The European Standards (EN 143 and EN 149) rank filtering facepiece (FFP) respirators as FFP1, FFP2, and FFP3, which are 80, 94, and 99% efficient, respectively, indicated by CE (for Conformité Européene) on complying products. They are tested with non-neutralized NaCl at 95 l/min.

**Figure 5:**
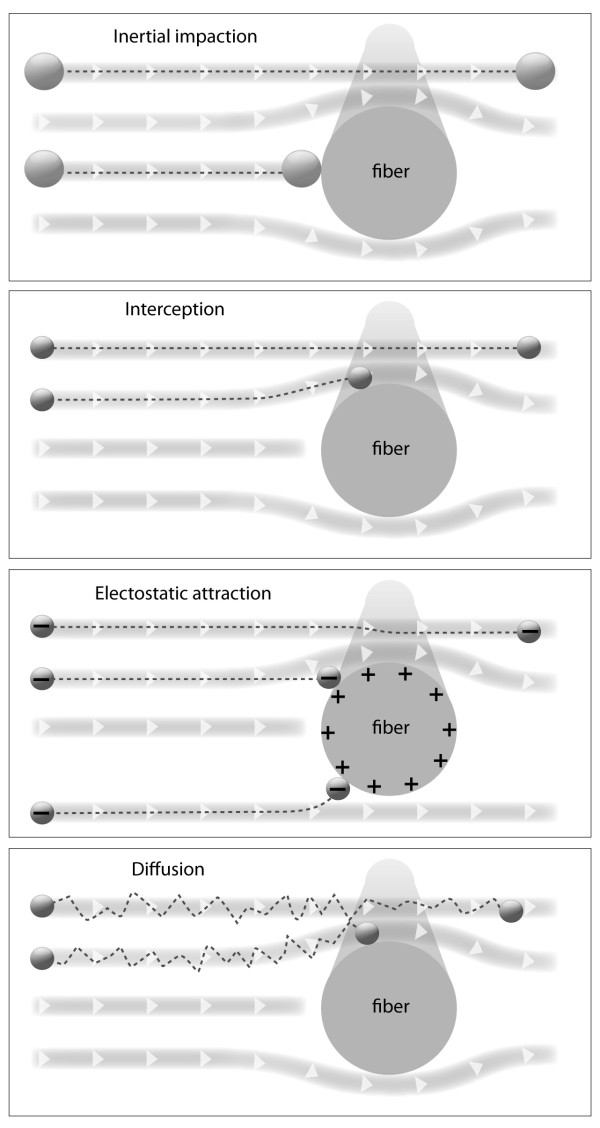
**The mechanisms of ENM association with fiber materials**. Each panel shows particles carried by airstreams, indicated by the bands with right pointing arrows. Some particles are retained by the fiber. Those that are not continue on the airstream past the fiber. The upper panel shows a large particle that is unable to follow the airstream around the fiber and collides with the fiber due to inertial impaction. The particle trapped by interception comes close enough to the fiber (within the particle radius) that it is captured by the fiber. Electrostatic attraction is discussed in the text **VI, C, 1. Respirators**. Small particles collide with each other, gas molecules, and other suspended matter in the air stream, resulting in Brownian motion and a random zigzagging path of movement, which may cause the particle to hit the fiber, as shown in the diffusion panel.

Particles > 100 nm are collected on filter media by two mechanisms: 1) inertial impaction in which air flow deviates around the fiber but large denser-than-air particles do not and impact the fiber due to their inertia, as shown in Figure 5; and 2) interception where the particle trajectory takes it within a particle radius of the fiber, which captures the particle. Airborne nanoparticles behave much like gas particles. Particles < 100 nm are collected by diffusion. Charged particles are trapped by electrostatic attraction, which involves an electrically charged particle and an electrically charged (electret) fiber. Electret filters are constructed from charged fibers. This appears to be a significant mechanism for respirator trapping of ENMs [210]. Neutral particles that pass through a charged fiber can be polarized by the electric field, thereby inducing charge to the particle. In dry conditions, ENM penetration decreases with time. With continued use, however, ENM penetration through an electrostatic filter increases; this was suggested to be due to the humidity of exhalation [211]. Soaking fiber filters in isopropanol removes electrostatic charge. Studies treating filtering facepiece respirators with isopropanol, and then drying them, showed increased penetration of particles > 30 nm [210], indicating electrostatic charge is a significant mechanism of fiber entrapment of ENMs above this size.

**Figure 6:**
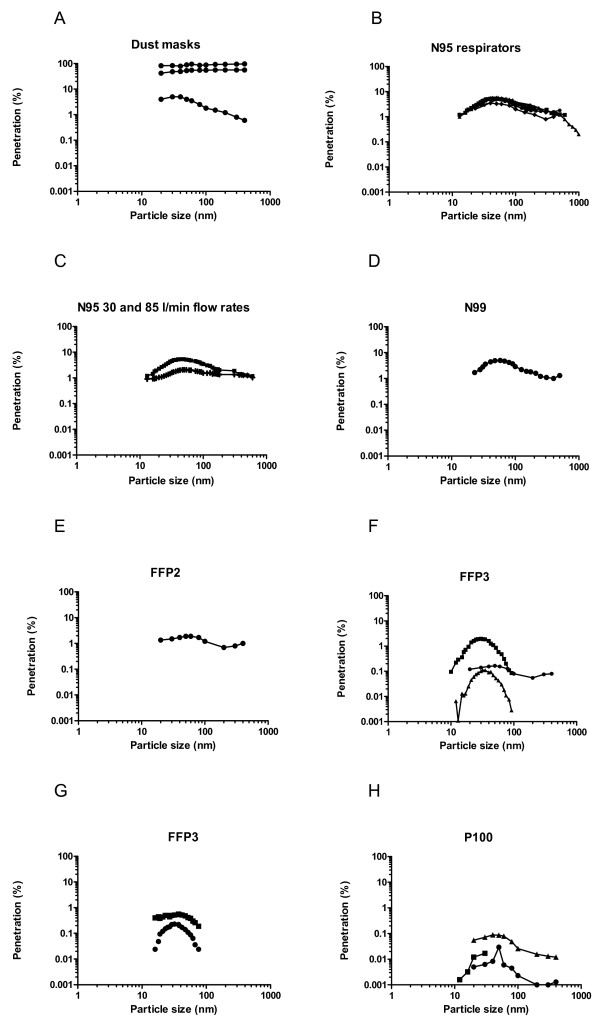
**Particle penetration through dust masks and facepiece respirators**. Test material was NaCl, flow rate 85 l/min and values shown are mean, unless noted otherwise. **Panel A**: **Dust masks**. Results shown are the mean and most and least efficient of 7 commercially available dust masks, as purchased in home improvement/hardware stores [225]. **Panel B**. **N95 respirators**. (Circle) Results from 6 3M Engineered nanoparticles and particulate respirators [http://multimedia.3m.com/mws/mediawebserver?mwsId=66666UuZjcFSLXTtN8T_NXM2EVuQEcuZgVs6EVs6E666666--]. (Square) Results from n = 2 [226]. (Triangle) Results from n = 1 at face velocity of 8.6 cm/sec [210]. (Diamond) Results from n = 5 [227]. (Hexagon) Results from n = 1 [212]. **Panel C**. **N95 respirators at two flow rates**. Results from n = 2 [226]. **Panel D. N99 respirators**. Results from n = 2 [212]. **Panel E. FFP2 respirators**. Results from n = 2 [228]. **Panel F. FFP3 respirators**. (Circle) Results from n = 1 [228]. (Square) Results from n = 1, with graphite at a face velocity of 9.6 cm/sec, flow rate not reported [211]. (Triangle) Results from n = 1, with graphite at a face velocity of 5.3 cm/sec, flow rate not reported [211]. **Panel G. FFP3 respirators**. (Circle) Results from n = 1, with graphite at face velocity of 5.3 cm/sec, flow rate not reported [217]. (Square) Results from n = 1, with titania at face velocity of 5.3 cm/sec, flow rate not reported [217]. **Panel H. P100 respirators**. (Square) Results from n = 2 with silver particles [229]. (Triangle) Results from n = 2 with NaCl [229]. (Circle) Results from n = 2 with NaCl [228].

Figure 6 shows results of some studies that assessed the efficacy of different types of dust masks and filtering facepiece respirators to retain ENMs. Most of the studies were conducted with different sizes of NaCl, but a few used silver, graphite or titania. The results show that dust masks purchased at hardware or home improvement stores would not be expected to protect the wearer. The results also show that the NIOSH and CE respirators generally limit penetration of ENMs to concentrations below their ranked efficiency level, which is based on 300 nm particles, except for the N99 filter, which did not retain more than 99% of nanoscale NaCl (Panel D). Most of the results shown were obtained with a flow rate of 85 l/min (modeling heavy work load). Flow rate affects particle penetration; an example is shown in Panel C where 30 l/min (modeling low/moderate intensity work) and 85 l/min flow rates were compared. Increasing flow rate increased penetration. This was further shown in a comparison of 30, 85, and 150 l/min flow rates with N95 and N99 filtering facepiece respirators [212], which is not shown in Figure 6. This highest flow rate was intended to model an instantaneous peak inspiratory flow during moderate to strenuous work. A similar result of ENM penetration positively correlating with air flow rate is shown in Panel F, where 5.3 and 9.6 cm/sec face velocity rates were compared.

The results shown in Figure 6 also indicate ENM penetration is influenced by particle composition. Panel G shows greater penetration of titania than graphite through FFP3 respirators under the same experimental conditions. Panel H shows greater penetration of 20 to 30 nm NaCl than silver ENMs of the same size through P100 filters. These results suggest further work is warranted to understand the influence of the physico-chemical properties of ENMs, particularly size, charge, and shape, on their penetration through filtering facepiece respirators. An issue that significantly impacts filtering facepiece respirator effectiveness is its seal around the face. "The biggest source of leakage is around the respirator seal because of poor fit" (Ronald E. Shaffer quoted in [213]). It has been estimated that 20% or more leakage occurs in respirators that are not properly fitted [214]. This underscores the importance of a proper fit for face mask respirators.

There is a particle size that maximally penetrates each filter material; the most penetrating particle size (MPPS). The results shown in Figure 6 indicate that the MPPS is ~40 to 50 nm for ENMs. This is approximately the same size of spherical ENMs that appear to contribute to their greatest differences in biological systems from solution and bulk forms of the same materials, as discussed in **II, A, 2. The physico-chemical properties of ENMs that impact their uptake**. This feature raises concern because the size of ENMs that may have the greatest effects in people are those that are best able to penetrate filtering facepiece respirators.

Until results are obtained from clinical-laboratory or work-place studies, traditional respirator selection guidelines should be used. These are based on OSHA Assigned Protection Factor values (the workplace level of respiratory protection that a respirator is expected to provide), the British Standards Institution Guide to implementing an effective respiratory protective device programme (BS 4275), and BS EN 529:2005 (Respiratory protective devices. Recommendations for selection, use, care and maintenance. Guidance document).

#### 2. Protective clothing

No guidelines are available on the selection of clothing or other apparel (e.g., gloves) for the prevention of dermal exposure to ENMs. This is due in part to the minimal data available on the efficacy of existing protective clothing, including gloves. Penetration of 10 to 1000 nm NaCl through woven and fibrous fabrics showed a MPPS between 100 and 500 nm and maximum penetration of 50 to 80% [215]. Comparison of graphite nanoparticle penetration through 650 μm thick cotton, 320 μm polypropylene, and 115 μm non-woven high-density polyethylene textile (Tyvek^®^) showed ~30, 12, and 4% penetration of the MPPS (~40 nm), respectively [216]. Tyvek^® ^permitted ~3 orders of magnitude less penetration of ~10 nm titania and platinum than cotton or a 160 μm woven polyester [217]. A study of ten nonwoven fabrics under conditions simulating workplace ENM exposure showed penetration increased with increasing air velocity and particle size (to ~300 to 500 nm). Pore structure of the various fabrics greatly influenced penetration [218]. Although nonwoven fabrics were much more effective to protect workers from ENM exposure than woven fabrics, they are much less comfortable to wear, suggesting improvements in fabric design or selection are needed to address this disincentive to use more effective PPE. The selection of laboratory coat materials can greatly influence the potential penetration of ENMs, which may end up on or penetrating street clothing, resulting in worker absorption or their even greater dispersion into the environment.

#### 3. Gloves

An unpublished study reported in 2005 the interaction of alumina and organoclay ENMs with powder-free (natural rubber) latex, powder-free (synthetic latex) nitrile, and cotton gloves [219]. Scanning electron microscopy showed that latex and nitrile gloves exhibited micrometer-sized surface pores/intrinsic voids. Although these surface imperfections were not complete holes, they may serve as pathways for the penetration of nanoparticles under unfavorable conditions, such as stretching and tearing. Stretching the latex and nitrile gloves to 200% of their original size greatly increased the pores/intrinsic voids. The surface pores may be important if they collect nanoparticles and the user does not remove the gloves when going to another location, thereby transporting the ENMs. Not surprisingly, there were wider gaps between the fibers in cotton gloves. The authors pointed out that ENMs may be treated with special coatings to enhance their dispersion characteristics, which may alter their permeability through glove materials. This study, however, did not determine the penetration of ENMs through gloves.

Nitrile, latex, and neoprene gloves prevented ~10 nm titania and platinum ENM penetration [217]. Double gloving has been suggested [219], which should reduce material penetration when there is glove perforation as well as dermal contamination when removing a contaminated outer glove. However, double gloving has not been shown to significantly decrease material penetration [220].

### D. Biological monitoring and medical examination

Secondary prevention in the continuum of the prevention and heirarchy of exposure control (Figure 4) includes biological monitoring and medical examination, the early detection of asymptomatic disease, and prompt intervention when the disease is preventable or more easily treatable [221]. Occupational health surveillance is the process by which information obtained from any activity in the continuum of prevention and heirarchy of exposure control is collected and used to support or alter what is done at a step higher in the heirarchy, as shown in the right upward pointing arrow in Figure 4 and discussed in [194]. Occupational health surveillance is the ongoing systematic collection, analysis, and dissemination of exposure and health data on groups of workers for the purpose of early detection and injury. It includes hazard surveillence, the periodic identification of potentially hazardous practices or exposures in the workplace, assessing the extent to which they can be linked to workers, the effectiveness of controls, and the reliability of exposure measures. A goal is to help define effective elements of the risk management program for exposed workers. Occupational health surveillance also includes medical surveillance, which examines health status to determine whether an employee is able to perform essential job functions [222]. It is required when there is exposure to a specific workplace hazard (OSHA, 29 CFR 1910.1450). This is different than medical screening or monitoring, a form of medical surveillance designed to detect early signs of work-related illness by administering tests to apparently healthy persons to detect those with early stages of disease or those at risk of disease. NIOSH concluded: "Currently there is insufficient scientific and medical evidence to recommend the specifc medical screening of workers potentially exposed to engineered nanoparticles" [222].

### E. Diagnosis, therapy, and rehabilitation

The third level in the continuum of prevention and heirarchy of exposure control, tertiary prevention, includes diagnosis, therapy, and rehabilitation. Owing to the lack of documented episodes of ENM exposure in humans that have resulted in adverse outcome, there is little experience with treatments of ENM exposure. One example that illustrates clever application of the knowledge of ENM properties was the use of UV light to visualize and treat the accidental dermal exposure of a human to quantum dots suspended in solution [223].

#### Good work practices

Based on the current knowledge of ENM exposure risks, some good workplace practices have been suggested. They are shown in Appendix 1.

#### An example of risk analysis and implementation of actions to limit ENM exposure

A recent study applied the principles of the Risk Assessment/Risk Management framework to identify and evaluate the potential hazards in a facility manufacturing ENMs [224]. The investigators established a measure of risk for each potential hazard and suggested improvement actions. These were then addressed with administrative controls, environmental monitoring, PPE and good workplace practices.

#### Some published guidelines for safe handling and use of ENMs

The following are some published guidelines, not regulations, for safe handling and use of ENMs. The Bundesanstalt für Arbeitsschutz und Arbeitsmedizin (BAUA) provided a "Guidance for handling and use of nanomaterials in the workplace" in 2007 [http://www.baua.de/en/Topics-from-A-to-Z/Hazardous-Substances/Nanotechnology/pdf/guidance.pdf;jsessionid=E81EECA3E6B5AD1A0D3D2396C4220AF5.2_cid137?__blob=publicationFile&v=2]. The Environmental Health and Safety office at the University of California provided "Nanotechnology: Guidelines for safe research practices" as their Safety Net #132 guidelines [http://safetyservices.ucdavis.edu/safetynets/Safetynets-Master%20List/Safetynets-Master%20List/safetynet-132-nanotechnology-guidelines-for-safe-research-practices]. Similarly, the Office of Environment, Health & Safety at the University of California prepared "Nanotechnology: Guidelines for Safe Research Practices" as their publication for the Berkeley Campus, Publication No. 73 [http://nano.berkeley.edu/research/73nanotech.pdf]. The Department of Energy, Nanoscale Science Research Centers, updated their "Approach to nanomaterial ES&H" in May 2008, as Revision 3a [http://orise.orau.gov/ihos/nanotechnology/files/NSRCMay12.pdf]. The Institute de recherche Robert-Sauvé en santé et en securité du travail (IRSST) published "Health Effects of Nanoparticles" [http://www.irsst.qc.ca/files/documents/PubIRSST/R-469.pdf].

The Environmental Health and Safety office of Massachusetts Institute of Technology prepared "Nanomaterials Toxicity", which is available at [http://ehs.mit.edu/site/content/nanomaterials-toxicity]. NIOSH has made available "Approaches to Safe Nanotechnology. Managing the Health and Safety Concerns Associated with Engineered Nanomaterials" [http://www.cdc.gov/niosh/topics/nanotech/safenano/]. The British Standards Institute prepared **"**Nanotechnologies - Part 2. Guide to safe handling and disposal of manufactured nanomaterials" in 2007, as their publication PD 6699-2:2007, ICS Number Code 13.100: 71.100.99 [http://www.nanointeract.net/x/file/PD6699-2-safeHandling-Disposal.pdf]. The American Society for Testing and Materials prepared

A "Standard Guide for Handling Unbound Engineered Nanoparticles in Occupational Settings", as their publication ASTM E2535-07 [http://www.astm.org/Standards/E2535.htm]. This is a guide for use when no specific information on ENMs or toxicity is available. OSHA prepared "Occupational exposure to hazardous chemicals in laboratories", as their publication 1910.1450 [http://www.osha.gov/pls/oshaweb/owadisp.show_document?p_table=standards&p_id=10106]. This guidance is designed for lab scale (i.e., not industrial) workers.

The Center for High-Rate Nanomanufacturing and NIOSH are preparing a guide to safe practices for working with nanomaterials that is anticipated to be released in early 2011.

Some websites that have considerable information on nanoscale materials are Nano Safe at [http://www.nanosafe.org], The International Council on Nanotechnology (ICON) [http://icon.rice.edu/], and "The GoodNanoGuide" [http://goodnanoguide.org/tiki-index.php?page=HomePage].

## Conclusions

An extensive variety of ENMs has been created. ENMs have already been utilized in many products and much more extensive use is anticipated in the future. There are reports of toxicity following *in vitro *and *in vivo *exposure to many ENMs, albeit often after very high doses, and generally lacking dose-response assessment. There is a small amount of exposure assessment information, and a paucity of information required for a risk characterization. Until more research and workplace monitoring information becomes available to refine the current knowledge of ENM risks, good workplace practices and guidelines based on ultrafine materials are guiding the occupational safety and health practices in working with ENMs.

## Appendix 1. Some good workplace practices

- Post signs indicating potential hazards, PPE requirements, and administrative controls at entrances to areas where ENMs are handled.

- Use appropriate PPE as a precaution whenever failure of a control, such as an engineering control, could result in ENM exposure, or ensure that engineering controls notify workers (e.g., alarms) when equipment malfunctions. Appropriate clothing and PPE generally includes closed-toed shoes, long pants without cuffs, long-sleeved shirt, laboratory coat, nitrile gloves, eye protection and perhaps a respirator, e.g., a half-mask P-100 or one that provides a higher level of protection, as appropriate to the ENM.

- Transfer ENMs between workstations in closed, labeled containers.

- Avoid handling ENMs in the open air in a 'free particle" state.

- Store dispersible ENMs, suspended in liquids or in a dry particle form, in closed (tightly sealed) containers whenever possible.

- Clean work areas potentially contaminated with ENMs at the end of each work shift, at a minimum, using either a HEPA-filtered vacuum cleaner or wet wiping methods. Do not dry sweep or use compressed air.

- Consider the use of disposable absorbent bench top coverings and laboratory coats.

- Place sticky floor mat at exit.

- Provide facilities for hand-washing, showering and changing clothes

Prohibit food, beverages and smoking in the work area.

## Competing interests

The authors declare that they have no competing interests.

## Authors' contributions

RAY initiated this review and created a draft of the manuscript that was discussed with RCM. RCM provided significant guidance on the structure of the review and its content, wrote the Risk Assessment/Risk Management framework section, integrated that section with the rest of the review, and provided much editorial guidance as RAY and RCM re-wrote and finalized the review. Both authors contributed to the revision of the review, responding to the reviewers' comments. Both authors read and approved the final manuscript.
